# Fertility drugs, reproductive strategies and ovarian cancer risk

**DOI:** 10.1186/1757-2215-7-51

**Published:** 2014-05-08

**Authors:** Federica Tomao, Giuseppe Lo Russo, Gian Paolo Spinelli, Valeria Stati, Alessandra Anna Prete, Natalie Prinzi, Marsela Sinjari, Patrizia Vici, Anselmo Papa, Maria Stefania Chiotti, Pierluigi Benedetti Panici, Silverio Tomao

**Affiliations:** 1 Department of Gynaecological and Obstetrical Sciences and Urological Sciences, University of Rome “Sapienza” Viale Regina Elena 324, 00161 Rome, Italy; 2 Department of Medico-Surgical Sciences and Biotechnologies, University of Rome “Sapienza,” Corso della Repubblica, 04100 Latina, Italy; 3 Department of Experimental Medicine, University of Rome “Sapienza”, Viale Regina Elena 324, 00161 Rome, Italy; 4 Department of Medical Oncology, National Cancer Institute of Rome, Italy, Rome

**Keywords:** Fertility drugs, Ovarian cancer, Ovarian stimulation, *in vitro* fertilization, Clomiphene citrate

## Abstract

Several adverse effects have been related to infertility treatments, such as cancer development. In particular, the relationship between infertility, reproductive strategies, and risk of gynecological cancers has aroused much interest in recent years. The evaluation of cancer risk among women treated for infertility is very complex, mainly because of many factors that can contribute to occurrence of cancer in these patients (including parity status). This article addresses the possible association between the use of fertility treatments and the risk of ovarian cancer, through a scrupulous search of the literature published thus far in this field. Our principal objective was to give more conclusive answers on the question whether the use of fertility drug significantly increases ovarian cancer risk. Our analysis focused on the different types of drugs and different treatment schedules used. This study provides additional insights regarding the long-term relationships between fertility drugs and risk of ovarian cancer.

## Introduction

In the world the number of people with problems of infertility has increased since 1990, resulting in a consistent increase in the use of strategies to improve fertility and reproductive rates. The highest incidence of infertility was found in western countries and in these countries a consistent proportion of the infertile women receive fertility treatments [1,2]. Moreover, the clinical use of fertility drugs and other reproductive strategies is expected to increase for the large number of women who postpone pregnancy for economic and social reasons [1,2].

In recent years, a great interest has been addressed to a supposed correlation between infertility treatments and cancers development, mainly breast, uterus and ovarian cancer [3-6].

Infertility appears to increase itself the incidence of ovarian carcinoma, while the potential additional risk associated with the use of fertility drugs is still debated. From many years nulliparity constitutes an established risk factor for ovarian cancer [7,8]. Conversely, several case-control studies failed to detect a significant correlation between fertility drugs use and ovarian cancer risk [9-14]. The work conducted by Ness et al. [9], analyzed 8 case-control studies. Among nulliparous women the risk of ovarian cancer increased by 2.67-fold (95% confidence interval (CI): 1.91 - 3.74). Among nulligravid women, neither any fertility drug use (odds ratio (OR) 1.60; 95% CI: 0.90-2.87) nor more than 12 months of use (OR 1.54; 95% CI: 0.45-5.27) was associated with increased risk of ovarian cancer. Fertility drug use in nulliparous women was associated with borderline serous tumors (OR 2.43; 95% CI: 1.01-5.88) but not with invasive epithelial cancers. These data suggest a role for the infertile status, but not for fertility drugs in the risk of epithelial ovarian cancer.

On the other hand, as reported in other studies, ovulation-stimulating therapies seem to be related to an increased risk of epithelial or borberline ovarian cancer [15-17].

However the literature data regarding an hypothetic correlation between ovarian cancer and infertility treatments, are conflicting and hard to interpret. This can be due to several factors. For example many studies evaluate fertility schedules of treatment containing drugs used in the past. Furthermore many reports did not show an optimal control on potential confounding factors such as the small number of patients, the frequent retrospective nature of the studies, the difficulty to evaluate the role of other reproductive factors influencing ovarian cancer risk.

The aim of this review is to analyze in detail the most important papers published on this topic in recent years.

## Materials and methods

We performed a review of the scientific literature concerning the association between the use of fertility treatments and the risk of ovarian cancer. We searched digital databases including Pubmed, EMBASE and the Cochrane Library. The survey was carried out using keywords such as “infertility”, “ovarian stimulation”, “ ovarian cancer risk”, “gynecological cancer”, “gynecological cancer risk”, “gonadotropins” , “human chorionic gonadotropin”, “clomiphene citrate”, “cancer risk”, “*in vitro* fertilization”, “progesterone”, “fertility drugs”, “infertility treatment” “gonadotropin-releasing hormone analogs”, variously associated together.

No period, language or study design restrictions have been applied in this stage of research. Reference lists of the most important papers were also examined and several authors were contacted by e-mail for more information about their work. The majority of the studies was excluded according to the title and to the content of the abstract.

In this review we did not include case reports and case series. Moreover were excluded studies exclusively assessing the fertility preservation after cancer treatment; also in vitro reports or animal studies were excluded. We analyzed the full versions of all relevant studies. We evaluated the selected information with a particular attention to the relationship between the occurrence of ovarian cancer and the treatments with fertility drugs. In particular we have focused our attention on the sample size, the type of infertility treatment regimens used, the time of follow-up and on the number of ovarian cancer reported.

## Results

Using the search criteria described in the previous section, we examined 970 papers and excluded 843 as irrelevant, on the basis of the title and abstract. The remaining 127 studies were considered in their full versions. Of these works, 97 were literature reviews or meta-analysis reports, 11 were case-control studies and 19 were cohort studies. The case-control studies often have been limited by the small number of subjects reporting prior drug use; therefore only some of these studies and the related meta-analysis have been discussed in this review. The 19 cohort studies selected are described in the Table 1, Table 2 and in the text. Just some meta-analyses, considered as the most significant, are extensively discussed in the text.

**Table 1:** **Fertility drugs and ovarian cancer** (**Cohort studies**)

**Study**	**Treatments**	**Population**	**Results**
**Rossing et al.**[21]**1994**	Clomiphene citrate	3837 women, 9 ovarian cancer in exposed, 2 ovarian cancer in unexposed	≥ 12 cycles with clomiphene citrate associated with RR = 11.1 (95% CI: 1.5-82.3) compared to the general population
**Potashnik et al.**[25] 1999	Definited as use of fertility drugs	1197 women. 1 ovarian cancer in exposed; 1 ovarian cancers in unexposed	SIR in exposed = 0.68 (95% CI: 0.01-3.80). SIR in unexposed = 1.35 (95% CI: 0.02-7.49).
**Doyle et al.**[23] 2002	Clomiphene citrate, hMG, hCG, GnRH analog,	4188 women, 4 ovarian cancers in exposed, 2 ovarian cancers in unexposed	SIR in exposed = 0.84 (95% CI: 0.23-2.15). SIR in unexposed = 1.67 (95% CI: 0.20-6.05). RR exposed vs unexposed = 0.59 (95% CI: 0,12-3,00)
**Brinton et al.**[26] 2004	Clomiphene citrate or gonadotropins	12193 infertile women, 15 ovarian cancers in exposed, 30 cancers in unexponed	RR exposed vs unexposed = 0.82 (95% CI: 0.4-1.5)
**Calderon-Margalit et al.**[24] 2009	Self reported exposure to fertility drugs	15030 parous women. Only 1 cancer in exposed 42 cancers in unexposed	No association found between fertility drugs and ovarian cancer (age-adjusted HR = 0.61). Only parous women
**Jensen et al.**[28] 2009	hMG, FSH, Clomiphene citrate, hCG, GnRH-analog,	54362 women, 156 ovarian cancers, 58 ovarian cancers in exposed, 98 cancers in unexponed	No risk increase associated with hMG, FSH, hCG, GnRH-analog. RR exposed vs unexposed for Clomiphene citrate: 1.14 (95% CI: 0.79- 1.64)
**Dos Santos Silva et al.**[29] 2009	Definited as use of fertility drugs	7355 women 12 cancers in exposed, 8 cancers in unexposed	SIR in exposed =1.10 (95% CI: 0,57-1.93) SIR in unexposed =0,78 (95% CI: 0.34-1.53) RR exposed vs unexposed =1,42 (95% CI: 0,53-3.99)
**Sanner et al.**[22] 2009	Clomiphene citrate and/or gonadotropins	2768 women, 16 cancers in exposed (9 ovarian cancers, 7 borderline tumors); 13 cancers in unexposed	SIR = 5.89 for ovarian cancer (95% CI: 1.91-13.75) SIR = 3.61 for borderline tumors (95% CI: 1.45-7.44). RR = 5.28 (95% CI: 1.70-16.47) for invasive cancers associated with gonadotropins
**Lerner-Geva et al.**[35]**2012**	Gonadotropins	2431 women, 18 ovarian cancer cases, 30 years of follow-up	SIR = 1.0 (95% CI: 0.59-1.57)
**Trabert et al.**[27] 2013	Clomiphene citrate, with or without gonadotropins	9825 women, 85 ovarian cancers	RR for clomiphene citrate = 1.34 (95% CI: 0.86-2.07) RR for gonadotropins = 1.00 (95% CI: 0.48-2.08)

*Abbreviations:RR* = relative risk, *CI* = confidence interval, *SIR* = standardized index ratio, *hMG* = human menopausal gonadotropin, *hCG* = human chorionic gonadotropin, *GnRH* = gonadotropin releasing hormone, *HR* = hazard ratio, *FSH* = follicle stimulating hormone.

**Table 2:** **IVF and ovarian cancer** (**Cohort studies**)

**Study**	**Treatments**	**Population**	**Results**
**Venn et al.**[44] 1995	IVF	29666 women, 3 cancers in exposed, 3 cancers in unexposed	SIR in exposed = 1.7 (CI 95%: 0.55-5.27) SIR in unexposed = 1.62 (95% CI: 0.52-5.02) RR exposed vs unexposed = 1,45 (95% CI: 0.28-7.55)
**Venn et al.**[45] 1999	IVF	29700 women, 7 ovarian cancers in exposed, 6 in unexposed	SIR in exposed = 0,88 (95% CI: 0,42- 1.84) SIR in unexposed = 1.16 (95% CI: 0.52-2.59)
**Dor et al.**[47] 2002	IVF	Retrospective cohort of 5026 women, 1 ovarian cancer case	SIR in exposed = 0.57 (95% CI: 0.01-3.20)
**Klip et al.**[48] 2002	IVF	23592 women, 17 ovarian cancers	No differences in risk exposed vs unexposed Detailed information obtained through questionnaires and from medical records
**Lerner Geva et al.**[43] 2003	IVF	1082 women, 3 ovarian cancers	SIR in exposed = 5.0 (95% CI: 1.02-14.6) SIR = 1.67 (0.02-9.27) when cancers developing within 1 year were excluded No untreated group Registry match
**Kallen et al.**[46] 2011	IVF	24058 women, 26 ovarian cancers	RR exposed vs unexposed = 2.09 (95% CI: 1,39-3.12)
**van Leeuwen et al.**[49] 2011	IVF	19146 IVF women, 6006 subfertile women not treated with IVF	Risk of borderline ovarian tumours increased in the IVF group compared with the general population. SIR = 1.76 (95% CI: 1.16-2.56). The overall SIR for invasive ovarian cancer was not significantly elevated, but increased with longer follow-up after first IVF. SIR = 3.54 (95% CI: 1.62-6.72) after 15 years.
**Yli**-**kuha et al.**[50] 2013	IVF	9175 women, 9 invasive ovarian cancers, 4 borderline ovarian tumors	OR for invasive cancers = 2.57 (95% CI: 0.69-9.23) OR for borderline tumors = 1.68 (95% CI: 0.31-9.27)
**Brinton et al.**[51] 2013	IVF	87403 women, 45 ovarian cancers	Global HR =1.58 (95% CI: 0.75-3.29), HR among women receiving ≥ 4 IVF cycles =1.78 95% CI: 0.76-4.13).

*Abbreviations:IVF* = in vitro fertilization, *SIR* = standardized index ratio, *CI* = confidence interval, *RR* = relative risk, *OR* = odds ratio, *HR* = hazard ratio.

### Clomiphene citrate and risk of ovarian cancer

Clomiphene citrate (CC) was used since the 1960s and is still considered one of the most important agents for women with anovulatory infertility; the drug have extensively showed to be able to reverse oligoovulation or anovulation in different reproductive pathologies; furthermore this agent was used, alone or in association with other agents, to induce ovarian hyperstimulation for *in vitro* fertilization (IVF) procedures [18,19].

This agent is a selective estrogen receptor modulator (SERM) that increases both estradiol and progesterone levels [20] and it is also able to increase cell proliferation; thus, an association between the use of CC and the risk of cancer has been hypothesized for gynecologic tumors, such as breast, ovarian cancer and endometrial cancer [3-6]. In the last years many authors investigated the relationship between ovarian cancer occurrence and the medical treatment with fertility drugs. Unfortunately all studies failed to give a definitive answer to the question. The causes of this conflicting result are several. We will discuss more in detail some of these studies with specific attention to the use of CC in the treatment of infertility and its association with ovarian cancer (Table 1).

Rossing et al. [21] evaluated the development of ovarian cancer (and in particular ovarian epithelial tumors) in a cohort study of 3837 women. There were 11 invasive or borderline malignant ovarian tumors, as compared with an expected number of 4.4 (standardized incidence ratio (SIR) 2.5; 95% CI: 1.3 - 4.5). Nine of the women in whom ovarian cancer developed were treated with CC; the adjusted relative risk (RR) among these women, as compared with infertile women who had not treated with this drug, was 2.3 (95% CI: 0.5-11.4). Five of the nine women had taken CC during 12 or more monthly cycles. This period of treatment was associated with an increased risk of ovarian tumors (RR 11.1; 95% CI: 1.5-82.3), whereas treatment with the drug for less than one year was not associated with an increased risk.

Similar results were reached by Sanner et al. [22]. They evaluated the incidence of ovarian cancer in a cohort of 2780 patients who received CC or gonadotropins. Also in women with gonadotropin treatment for non-ovulatory disorders, the risk was elevated (SIR 5.89; 95% CI: 1.91-13.75) but 4 of the 5 cases reported human Chorionic Gonadotropin (hCG) treatment only. A multivariate analysis indicated that treatment with gonadotropins only was associated with an increased risk of invasive cancer (RR 5.28; 95% CI: 1.70-16.47). For borderline tumors, a more than threefold overall increase of tumors (SIR 3.61; 95% CI: 1.45-7.44) was observed; women exposed to CC, because of ovulatory disorders, showed the highest risk (SIR 7.47; 95% CI: 1.54-21.83).

Most of other investigations did not confirm a link between fertility drugs use and ovarian cancer risk [23-25].

In a retrospective study Brinton et al. [26] evaluated 12193 infertile women followed for a median of 18.8 years and reported 45 ovarian cancers. This study used a detailed collection of informations about drug exposures, causes of infertility, and other potential cancer risk factors. The results were largely reassuring, showing no risk increase associated with the use of either CC or gonadotropins. The recent published study by Trabert et al. [27] is actually a 30 year follow-up to the original study by Brinton et al. [26] and examined the association between the use of ovulation-inducing drugs and the risk of ovarian cancer in a retrospective cohort study of 9825 women. In this study an increase in ovarian cancer risk was not observed after an extensive use of CC (adjusted RR 1.34; 95% CI: 0.86-2.07) or gonadotropins (RR 1.00; 95% CI: 0.48-2.08), with the only exception of those patients who used CC and failed to become pregnant. In fact they had a higher risk than those who successfully conceived compared with nonusers (respectively, RR 3.63; 95% CI: 1.36-9.72 vs RR 0.88; 95% CI: 0.47-1.63). Despite these results, the reason for an association between CC use and ovarian cancer risk among persistently nulligravid women was not clearly determined.

Jensen et al. [28] identified 156 ovarian cancer cases, through a linkage with the Danish Cancer Registry. The authors did not suggest an increased ovarian cancer risk associated with the use of gonadotropins (RR 0.83; 95% CI: 0.50-1.37), CC (RR 1.14; 95% CI: 0.79-1.64), hCG (RR 0.89; 95% CI: 0.62-1.29), or gonadotropin releasing hormone (GnRH) (RR 0.80; 95% CI: 0.42-1.51). Furthermore, no positive or negative associations were found considering all four groups of fertility drugs used, the number of cycles, the length of follow-up, or the rates of parity.

Dos Santos Silva et al. [29] identified 21 ovarian cancers among 7355 women followed for infertility for over 20 years, in order to assess long-term health effects of ovarian-stimulation drugs. They observed no significant differences in the risk of ovarian and other tumors in women treated or not treated with ovarian stimulating drugs.

### Other fertility drugs and risk of ovarian cancer

In the treatment of female infertility several drugs are now more spread than CC. GnRH analogues/agonists, human menopausal gonadotropin (hMG), progesterone, follicle stimulating hormone (FSH), luteinizing hormone (LH) and hCG are commonly used as single agents or in combination with CC. Moreover, several other associations among these different drugs have been tested or are under investigation [30-33].

We know that gonadotropins, hCG, progesterone, FSH and LH, have been recognized as growth factors in ovarian cancer. In a recent study Hilliard et al. [34] evaluated the pathways activated by FSH and LH in normal ovarian surface epithelium (OSE) growth. The purpose of this study was to identify the pathways downstream of the gonadotropins in normal OSE and their contribution towards proliferation and oncogenesis. The data obtained suggest that the gonadotropins stimulate some of the same proliferative pathways activated in normal OSE and in ovarian cancers too.

Due to the evidence that in the treatment of infertile women these different agents are commonly used together and in combination with CC, it is very difficult to evaluate separately the role of every single agent in development of ovarian cancer. For this reason we have analyzed in the previous section and in the Table 1 the majority of studies on this topic. Only two studies are discussed in detail on this chapter [35,36].

Lerner-Geva et al. [35] presented a study to evaluate the possible risk for cancer development in a cohort of 2431 women who were treated for infertility with gonadotropins and other fertility drugs in Israel, with over 30 years of follow-up. They calculated the SIR between the observed cancer cases and the expected cancer rates in the general population. The investigators observed 18 cases of ovarian tumors compared to 18.1 expected (SIR 1.0; 95% CI: 0.59-1.57). Ovarian cancer risk was not found to be elevated and the authors were not able to demonstrate a significant high risk associated with ovulation stimulating treatments.

In a recent work Rizzuto et al. [36] included 11 case-control studies and 14 cohort studies, for a total of 182972 women. They did not show an increased ovarian cancer risk in women exposed to CC alone or CC plus gonadotropin, compared with unexposed women. For borderline ovarian tumors, exposure to any fertility drug was associated with a two to three-fold increased risk in two case-control studies. One case-control study reported an OR of 2,8 (95% CI: 1.5-5.16), which was based on only 4 cases. In another cohort study, there was more than a two-fold increase in the incidence of borderline tumors compared with the general population (SIR 2.6; 95% CI: 1.4-4.6), while in another report a Hazard Ratio (HR) of 4.23 (95% CI: 1.25-14.33) for the risk of a borderline ovarian tumor was reported (subfertile treated women compared with non-treated group with more than one year of follow-up).

### IVF and risk of ovarian cancer

IVF is used for the treatment of all types of infertility. This is a medical technique by which an egg is fertilised by sperm outside the body. The fertilised egg (zygote) is then transferred into the patient's uterus with the intent to establish a successful pregnancy. IVF requires a pharmacological ovarian hyperstimolation. Generally the intensive ovulation induction treatments are represented by injectable gonadotropins (FSH analogues), GnRH agonist and GnRH antagonist [37-40].

Some studies suggested an association among the use of ovulation-inducing drugs, IVF, and ovarian cancer risk, but only few cases of ovarian cancer have been described in women followed in IVF programs [41,42]. Therefore the relationship between IVF and development of ovarian cancer is still under investigation (Table 2).

Lerner-Geva et al. [43] evaluated the association between ovarian hyperstimolation with IVF and an increased risk of cancer development, using a cohort of 1082 women, who were followed with a mean follow-up of 6.5 ± 2.2 years. They observed 21 cases of cancer as compared to the 11 expected (SIR 1.91; 95% CI: 1.18-2.91). These included 11 cases of gynecological tumors and in particular 3 cases of ovarian cancer as compared to 0.60 expected (SIR 5.0; 95% CI: 1.02-14.6). However SIR decreased to 1. 67 (95% CI: 0.02-9.27) while cases developing within 1 year were excluded; the authors concluded that the higher than expected cancer rate could not be attributed to IVF treatments.

Venn et al. in the first of two studies [44] observed 6 ovarian cancers, among 29666 women. For ovarian cancer SIRs were 1.70 (95% CI: 0.55-5.27) and 1.62 (95% CI: 0.52-5.02), respectively in exposed and unexposed women. In the second study Venn et al. [45] not confirmed these results. The cohort consisted of 29700 women: 20656 were exposed to fertility drugs and 9044 were not. Thirteen ovarian cancers occurred among these women. The incidence was no greater than expected in the exposed group (SIR 0.88; 95% CI: 0.74-1.13) and in unexposed group (SIR 1.16; 95% CI: 0.52-2.59). Women with unexplained infertility had significantly more ovarian cancers than expected (SIR 2.64; CI: 1.10-6.35).

Other contrasting results regarding IVF and ovarian cancer risk derive from a study conducted in Sweden and focused on cancer developing among women who gave birth following IVF treatment. In this experience Kallen et al. [46] found a significantly elevated risk of ovarian cancer following IVF treatments (RR 2.09; 95% CI: 1.39-3.12). Nevertheless many other investigators stressed the hypothesis that risk of ovarian cancer was not associated with effect of IVF [47,48]. In a cohort of 25152 women Klip et al. [48] reported 17 ovarian cancer and showed no difference in the risk of ovarian cancer between treated and untreated women.

On the other hand, in a recent study van Leeuwen et al. [49], identified a cohort of 19146 women who received IVF and a comparison group of 6006 sub-fertile women who were not treated with IVF. The incidence of ovarian malignancies was assessed through linkage with disease registries. The risk of ovarian malignancies in the IVF group was compared with the risk observed in the general population and in the sub-fertile comparison group. After a median follow-up of 14.7 years, the risk of borderline ovarian tumors was increased in the IVF group compared with the general population (SIR 1.76; 95% CI: 1.16-2.56). The overall SIR for invasive ovarian cancer was not significantly elevated, but increased when the follow-up was extended after first IVF (P = 0.02); the SIR reached 3.54 (95% CI: 1.62-6.72) after 15 years. The risk of borderline ovarian tumors and of all ovarian malignancies in the IVF group were significantly increased compared with the risk in the sub-fertile control group (HR 4.23; 95% CI: 1.25-14.33 and 2.14; 95% CI: 1.07-4.25, respectively, adjusted for age, parity and subfertility cause).

Yli-kuha et al. [50] compared cancer risk among patients receiving IVF with that found in the general population. During the follow-up period after IVF, the investigators observed 9 (OR 2.57; 95% CI: 0.69-9.23) invasive ovarian cancers and 4 (OR 1.68; 95% CI: 0.31-9.27) borderline ovarian tumors. These results confirmed that IVF women had three times more invasive ovarian cancers than controls (only three case about 9175 women), but this difference was not statistically significant. The limitations of this study were: the small number of cases, the absence of subgroups and the very limited information about the different drugs used and their dosages.

Brinton et al. [51] also evaluated long-term cancer risk associated with IVF, calculating HRs for different gynecological cancers. The investigators included in their study a total of 87403 women treated for infertility on or after September 1994, who were followed for cancer development through June 2011. Only 45 ovarian cancers were identified. So they did not find a significant relationship between IVF technique and gynecological cancer risk. However, compared with women with no fertility treatment, the HR for ovarian cancer associated with IVF was 1.58 (95% CI: 0.75-3.29), with higher risk among those receiving ≥ 4 IVF cycles (HR 1.78; 95% CI: 0.76-4.13). The authors concluded that women receiving this treatment should continue to be monitored during the years.

Two recent meta-analyses have recently been published on this topic [41,42]. In the study of Siristatidis et al. [41], nine cohort studies were analyzed (109969 women exposed to IVF with 76 cases of ovarian cancer). The comparison of studies, considering the general population as the reference group, found a statistically significant association between the use of IVF and an increased risk for ovarian cancer (RR 1.50; 95% CI:1.17-1.92). On the contrary, when infertile women were used as the reference group, no significant associations with ovarian cancer were noted (RR 1.26; 95% CI: 0.62-2.55). So IVF does not seem to be associated with elevated ovarian cancer risk when the confounding effect of infertility was neutralized. Of note, only one study provided follow-up longer than 10 years for the group exposed to IVF. In this meta-analysis borderline tumours were not included.

In the meta-analysis published by Li et al. in 2013 [42], eight cohort studies involving 746455 patients were included. In this work authors evaluated the association between IVF and all-site cancers and in particular observed a RR of 1.59 (95% CI:1.24-2.03) for ovarian cancer. A high risk of ovarian cancer was observed in the analysis of subgroups and especially in women who were diagnosed with cancer during or shortly after IVF (<1 year after treatment).

## Discussion

Several investigators explored the safety profile of fertility drugs and the risks associated with their use [52-57], (Tables 1 and 2). The results emerging from the studies included in our review are contrasting. Some works suggest the hypothesis that fertility drugs do not significantly contribute to ovarian cancer risk [9-14,23-29,35,41,43,45,47,48]. Other studies have reported an increased risk of ovarian cancer in women treated with fertility drugs [15-17,21,22,42,44,46,49-51]. Finally some studies have reported an increased risk especially for borderline ovarian tumors [16,21,22,36,42,49,50].

Establishing the correlation between fertility drugs use and ovarian cancer risk is complex because it is know that infertility itself determines an increased risk of cancer [7,8]. Three major theories have been proposed to explain the ovarian cancer pathogenesis [58-65]. The “Fallopian tube theory”, hypothesized by Kurman et al., suggested that serous ovarian carcinomas developed from normal residual fimbrial epithelium localized on the ovarian surface after ovulation. The author supposed that, following implantation of tubal epithelium in the ovary, the adjacent stromal cells are activated and secrete steroid hormones that can stimulate malignant transformation [58,59].

The “Incessant ovulation theory” hypothesizes that the frequent and repetitive trauma to the ovarian epithelium, caused during ovulation, contributes to DNA damage, increasing ovarian cancer risk. In nulliparous women this damage is incessant, so that DNA injuries are facilitated. This can lead to malignant cells transformation [60-63]. The last hypothesis is the “Gonadotropin theory”. It suggests that an increase in FSH and LH lead to an overstimulation of the ovarian epithelium by increasing local levels of estrogen. This plays an important role in ovarian cancer development. A support to this theory arises from the observation that ovarian cancer incidence increases considerably during menopause, when gonadotropin levels grow [64,65].

According to these three theories, fertility drugs should be related to an increase in ovarian cancer risk, because they can cause a gain in LH and FSH levels, and stimulate ovulation. But women who assume fertility drugs have *per se* a high risk because of their infertility [7,8]. It is clear that one of the main difficulties in this field is to separate these risk factors, presenting together in infertile women treated with fertility drugs.

Three large meta-analyses have been conducted about our issue [41,42,66]. Two of them [44,66] concluded that there was no difference in ovarian cancer risk between infertile women treated for their infertility and infertile not treated women. The third meta-analysis [44] show an increased risk of ovarian cancer in patients who have used fertility drugs.

We can conclude that past and recent scientific reports reached different results because these studies are characterized by some methodological limitations: low sample size; low follow-up period; low number of ovarian cancers reported; self-reported drugs assumption; lack of information on the type of drugs used, the dosage and the number of cycles administered; lack of attention to the other reproductive risk factors for ovarian cancer; lack of a clear distinction between epithelial tumors and borderline tumors.

Considering all the studies included in our review, the most recent works appear reassuring regarding the potential risk of ovarian cancer, and more accurate compared to the past, because they are conceived in order to avoid the interrelationships and potential bias derived from the different risk factors.

## Conclusions

In the next years, the incidence of female infertility is expected to increase. A lot of new drugs are under investigation while other recent drugs are already in current use, such as aromatase inhibitors [67-70]. Moreover preservation of fertility and reproduction in cancer patients, constitutes today an emerging problem in clinical oncology, and the new reproductive technologies begin to be used also in this group of patients [70,71]. These new drugs and technologies will need to be tested for their safety in the perspective of an hypothetic correlation with ovarian and gynaecological cancers development. New studies are expected to be designed differently from the past, in particular to reduce confounding factors. Furthermore, the new studies would look even at borderline ovarian tumors, because they are often not included in cancer registries or are improperly associated with other ovarian tumors.

Another crucial point is the improvement in knowledge about ovarian cancer and its pathogenesis. In fact the three main theories about ovarian cancer development seem to be equally plausible and not necessarily contradict each other.

This issue is fascinating and has a notable social impact.

## Abbreviations

CI: Confidence interval; OR: Odds ratio; CC: Clomiphene citrate; IVF: *in vitro* fertilization; SERM: Selective estrogen receptor modulator; SIR: Standardized incidence ratio; RR: Relative risk; hCG: Human chorionic gonadotropin; GnRH: Gonadotropin releasing hormone; hMG: Human menopausal gonadotropin; FSH: Follicle stimulating hormone; LH: Luteinizing hormone; OSE: Ovarian surface epithelium; HR: Hazard ratio.

## Competing interests

The authors have no relevant affiliations or financial involvement with any organization or entity with a financial interest in or financial conflict with the subject matter or materials discussed in the manuscript. This includes employment, consultancies, honoraria, stock ownership or options, expert testimony, grants or patents received or pending, or royalties. No writing assistance was utilized in the production of this manuscript.

## Authors’ contributions

FT, GLR, GPS, PBP, PV and ST have contributed to conception and design of review, acquisition, analysis and interpretation of data and to the drafting of manuscript; VS, AAP, NP, MS, AP, MSC have contributed to acquisition, analysis and interpretation of data and to the drafting of manuscript. All authors read and approved the final manuscript.

## Authors’ information

FT: MD phd, GLR: MD, GPS: MD, phd, VS: MD, AAP: MD, NP: MD, MS: MD, PV: MD, AP: MD, MSC: MD, PBP: MD, ST: MD.
